# Integrated TCR repertoire analysis and single-cell transcriptomic profiling of tumor-infiltrating T cells in renal cell carcinoma identifies shared and tumor-restricted expanded clones with unique phenotypes

**DOI:** 10.3389/fonc.2022.952252

**Published:** 2022-09-14

**Authors:** Yuexin Xu, Alicia J. Morales, Andrea M. H. Towlerton, Shreeram Akilesh, Chris P. Miller, Scott S. Tykodi, Edus H. Warren

**Affiliations:** ^1^ Clinical Research Division, Fred Hutchinson Cancer Center, Seattle, WA, United States; ^2^ Department of Laboratory Medicine and Pathology, University of Washington School of Medicine, Seattle, WA, United States; ^3^ Department of Medicine, Division of Medical Oncology, University of Washington School of Medicine, Seattle, WA, United States

**Keywords:** renal cell carcinoma, tumor-infiltrating T-cells, single-cell RNA sequencing, T-cell receptor repertoire, tumor microenvironment (TEM)

## Abstract

Objective responses of metastatic renal cell carcinoma (RCC) associated with systemic immunotherapies suggest the potential for T-cell-mediated tumor clearance. Recent analyses associate clonally expanded T cells present in the tumor at diagnosis with responses to immune checkpoint inhibitors (ICIs). To identify and further characterize tumor-associated, clonally expanded T cells, we characterized the density, spatial distribution, T-cell receptor (TCR) repertoire, and transcriptome of tumor-infiltrating T cells from 14 renal tumors at the time of resection and compared them with T cells in peripheral blood and normal adjacent kidney. Multiplex immunohistochemistry revealed that T-cell density was higher in clear cell RCC (ccRCC) than in other renal tumor histologies with spatially nonuniform T-cell hotspots and exclusion zones. TCR repertoire analysis also revealed increased clonal expansion in ccRCC tumors compared with non-clear cell histologies or normal tissues. Expanded T-cell clones were most frequently CD8^+^ with some detectable in peripheral blood or normal kidney and others found exclusively within the tumor. Divergent expression profiles for chemokine receptors and ligands and the Ki67 proliferation marker distinguished tumor-restricted T-cell clones from those also present in blood suggesting a distinct phenotype for subsets of clonally expanded T cells that also differed for upregulated markers of T-cell activation and exhaustion. Thus, our single-cell level stratification of clonally expanded tumor infiltrating T-cell subpopulations provides a framework for further analysis. Future studies will address the spatial orientation of these clonal subsets within tumors and their association with treatment outcomes for ICIs or other therapeutic modalities.

## Introduction

Despite the development of molecular targeted therapies including tyrosine kinase and mTOR inhibitors that have prolonged survival, metastatic renal cell carcinoma (RCC) continues to be an incurable disease for most patients. It has long been observed that clear cell RCC (ccRCC) can be sensitive to systemic cytokine-based immunotherapy. More recently, immune checkpoint blocking therapies, alone or in combination with targeted therapies, have shown significant clinical benefits for advanced ccRCC, firmly establishing immunotherapy as the preferred front-line treatment. However, complete radiographic responses are uncommon, occurring in only 6-16% of patients receiving immune checkpoint inhibitor (ICI)-containing combination regimens (1–4). The factors contributing to primary or acquired tumor resistance and heterogenous treatment outcomes are still poorly understood.

While ccRCC tumors have been shown to be among the most highly immune cell infiltrated tumor types, retrospective analyses before the introduction of ICI therapies found a negative association of immune cell infiltration and prognosis (5). Large scale retrospective tumor tissue-based analyses in association with clinical trials of ICI therapies for advanced or metastatic ccRCC have consistently identified markers of T-cell inflammation positively associated with treatment outcomes (6–8). However, cross trial validation of specific markers has remained inconclusive (7, 9).

Clonal expansion is a hallmark of activated T cells responding to their target antigen. Indeed, expression of the proliferation marker Ki67 on tumor-infiltrating CD8^+^ T cells is an independent favorable prognostic factor in RCC (10). Recent single-cell based T-cell receptor (TCR) profiling has focused attention on clonotypic T-cell expansions in the ccRCC tumor microenvironment (TME). These expanded clones adopt an exhausted phenotype in association with more advanced disease consistent with their recognition of putative tumor antigens (11, 12). The recent prospectively designed ADAPTeR study demonstrated that the abundance of clonally expanded CD8**^+^** T cells present in the tumor at diagnosis and their persistence during treatment with anti-PD1 therapy were predictors of response (13). This suggested that tumor antigen-reactive T cells within the tumor at diagnosis can be induced to overcome their exhausted phenotype with ICI therapy.

To identify and further characterize the clonally expanded T cells associated with renal tumors, we analyzed and report on the density, spatial distribution, TCR repertoire, and transcriptome of tumor-infiltrating T cells from 14 renal tumors and compared them with T cells present in peripheral blood and normal adjacent (kidney) tissue (NAT). Targeted and whole-transcriptome single-cell RNA sequencing (scRNAseq) identified previously uncharacterized phenotypic subsets of expanded tumor-infiltrating (T) lymphocyte (TIL) clones that are exclusive to tumor or present in blood, suggesting a newly recognized functional divergence.

## Materials and methods

### Study cohort

This research was approved by the Institutional Review Board at our center, and all patients provided written informed consent for donation of biological samples for research use. We collected 45 biospecimens of primary tumor, NAT, and peripheral blood mononuclear cells (PBMC) from 14 treatment naïve patients with renal tumors who underwent partial or full nephrectomy surgery. Routine clinical pathology assessment of the primary tumors revealed 10 ccRCC, one chromophobe RCC, one type I papillary RCC, one oncocytoma, and one sarcoma (**Table 1**).

**Table 1:** Study cohort clinic and pathologic features.

Demographics			Pathology							Experiment			
patient	age	sex	diagnosis	max diameter	pStage (TN)	grade	sarcomatoid	rhabdoid	necrosis	TRB-DNAseq	mIHC	targetedSC	10X-SC
1	37	M	ccRCC*	5.1 cm	pT3a, pN0	3	NS	NS	10%	Yes	Yes	No	No
2	70	M	ccRCC	6.0 cm	pT3, pNx	2	none	none	35%	Yes	Yes	Yes	No
3	58	F	oncocytoma	4.0 cm	NA	NA	NS	NS	NS	Yes	Yes	Yes	No
4	70	M	ccRCC	4.0 cm	pT1a, pNx	2	NS	NS	none	Yes**	Yes	Yes	No
5	74	M	ccRCC	13.5 cm	pT3b, pN0	4	40%	5%	30%	Yes	Yes	No	No
6	63	M	papillary, type I	7.8 cm	pT2, pNx	2	none	none	none	Yes	Yes	No	No
7	48	M	ccRCC	12.5 cm	pT3a, pN0	4	NS	NS	15%	Yes	Yes	Yes	No
8	62	F	ccRCC	7.2 cm	pT3a, pNx	2	none	none	none	Yes	Yes	Yes	No
9	73	F	pleomorphic sarcoma, high grade	9.5 cm	pT1, pN0	3 (FNCLCC)	NA	NA	20%	Yes	Yes	No	No
10	41	F	ccRCC	2.4 cm	pT1a, pNx	3	none	none	none	Yes	Yes	No	No
11	77	M	ccRCC	5.0 cm	pT3a, pNx	3	none	none	5%	Yes	Yes	Yes	No
12	44	M	chromophobe RCC	9.0 cm	pT2a, pNx	NA	none	none	none	Yes	Yes	No	Yes
13	66	M	ccRCC	7.6 cm	pT3a, pN0	2	none	none	none	Yes	Yes	No	Yes
14	46	F	ccRCC	3.0 cm	pT3a, pNx	3	none	none	none	Yes	Yes	No	Yes

NS, not stated; NAT, normal renal cortex; NA, not applicable.

*suspicion for translocation RCC without confirmatory molecular testing; Chromosomal Genomic Array Testing suggestive of ccRCC.

**TRB-seq was done on FFPE curls from tumor due to the limited sample size.

### Sample processing

Single-cell suspensions were prepared from primary tissues using the gentleMACS™ tissue dissociator and tumor dissociation kit (Miltenyi Biotec, Bergisch Gladbach, Germany) on the “soft tissue” setting. PBMC were processed using Lymphocyte Separation Medium (Corning, Corning, NY) by density gradient centrifugation or SepMate Isolation (StemCell Technologies, Vancouver, Canada). Sections of each tumor specimen were formalin-fixed and paraffin-embedded (FFPE) for subsequent analysis by immunohistochemistry (IHC).

### Multiplex IHC

The following antibodies were used to perform mIHC on FFPE samples: anti-human CD4, (clone SP35, Cell Marque, Rocklin, CA, dilution 1:100), anti-human CD3e (clone F7.2.38, Abcam, Eugene, OR, dilution 1:100), anti-human CD8 (clone EP334, Bio SB, Santa Barbara, CA, dilution 1:100), anti-human Carbonic anhydrase IX (CAIX) (clone TH22, Leica Biosystems, Buffalo Grove, IL, dilution 1:25), anti-human Ki67 (clone MIB1, Agilent Technologies, Santa Clara, CA, dilution 1:50). The experimental conditions for CAIX IHC were described previously (14). The staining was performed using the automated OPAL (PerkinElmer, Waltham, MA) workflow on the Leica Bond Rx staining platform (Leica Biosystems, Buffalo Grove, IL) by the Experimental Histopathology Core at our center. Briefly, formalin-fixed paraffin-embedded tissues were sectioned at 4 microns onto positively-charged slides and baked for 1 hour at 60°C. The slides were then dewaxed and stained on a Leica BOND Rx stainer (Leica, Buffalo Grove, IL) using Leica Bond reagents for dewaxing, antigen retrieval and antibody stripping. A high stringency wash was performed after the secondary and tertiary applications using high-salt TBST solution (0.05M Tris, 0.3M NaCl, and 0.1% Tween-20, pH 7.2-7.6). Leica’s PowerVision Poly-HRP anti-Rabbit Detection or Leica’s PowerVision Poly-HRP anti-Mouse Detection species specific polymer was used for all secondary applications.

Antigen retrieval and antibody stripping steps were performed at 100°C with all other steps at ambient temperature. Endogenous peroxidase was blocked with 3% H_2_O_2_ for 8 minutes followed by protein blocking with TCT buffer (0.05M Tris, 0.15M NaCl, 0.25% Casein, 0.1% Tween 20, pH 7.6 +/- 0.1) for 30 minutes. The first primary antibody was applied for 60 minutes followed by the secondary antibody application for 20 minutes and the application of the tertiary TSA-amplification reagent (OPAL fluor, PerkinElmer) for 20 minutes. The primary and secondary antibodies were stripped with retrieval solution for 20 minutes before repeating the process with the second primary antibody starting with a new application of 3% H_2_O_2_. The process was repeated until all 5 antibodies were applied. There was no stripping step after the 5^th^ antibody. Slides were removed from the stainer and stained with Spectral DAPI (PerkinElmer, Waltham, MA) for 5 minutes, rinsed for 5 minutes, and covered with Prolong Gold Antifade reagent (Invitrogen/Life Technologies, Grand Island, NY).

Slides were cured for 24 hours at room temperature, then whole slide images from each slide were acquired on a PerkinElmer Vectra 3.0 Automated Imaging System. Images were spectrally unmixed using PerkinElmer inForm software. Images were then analyzed with HALO image analysis software (Indica Labs, Albuquerque, NM). Individual cells were identified based on nuclear recognition (DAPI stain) and the imaging software then measured fluorescence intensity of the estimated cytoplasmic areas for each cell. A mean intensity threshold above background was used to determine positivity for each fluorochrome within the cytoplasm, thereby defining cells as either positive or negative for each marker. The positive cell data for the CAIX marker was then used to define tumor borders in ccRCC to facilitate nearest neighbor spatial analyses. For each sample, five to ten representative areas with continuous CAIX^+^ or CAIX^-^ cells from each sample were selected to train a classifier in HALO. The classifier was then applied to samples to define viable tumor areas *via* a whole slide scan. CAIX expression was visually confirmed within the tumor border after classification.

### TCR repertoire analysis

Genomic DNA was isolated from the tumor, NAT and PBMC single cell suspensions using QIAamp blood mini kits (Qiagen, Hilden, Germany). Targeted *TRB*-complementarity determining region 3 (*TRB-*CDR3) libraries were constructed using the ImmunoSEQ hsTCRB v3.0 kit (Adaptive Biotechnologies, Seattle, WA) on 1µg of genomic DNA isolated from the biospecimens according to the manufacturer’s protocol. Briefly, bias-controlled multiplex PCR was applied to amplify all possible rearranged genomic TCRβ sequences using an equimolar pool of the 45 TCR Vβ forward primers, and an equimolar pool of the TCR Jβ reverse primers. The following thermal cycling conditions were used for amplification: 1 cycle at 95°C for 15 minutes, 25 to 40 cycles at 94°C for 30 seconds, 59°C for 30 seconds, and 72°C for 1 minute, followed by 1 cycle at 72°C for 10 minutes (15). The final PCR products at 200bp length were pooled and sequenced at survey level resolution on the Illumina MiSeq platform (v3 150 cycle) in the Genomics Core Facility at the Fred Hutchinson Cancer Research Center as previously described (16, 17).

### scRNAseq

To prepare samples for scRNAseq, single-cell suspensions were thawed from cryogenic storage and stained with DAPI, 1:20 dilution of APC-Cy7-labeled anti-CD3 mAb (clone SK7; BD Biosciences, San Jose, CA), 1:20 dilution of FITC-labeled anti-CD8 mAb (clone RPA-T8; BD Biosciences), and 1:50 PE-labeled anti-CD45 mAb (clone HI30; BD Biosciences), and sorted by flow cytometry (BD FACSymphony™, BD Biosciences). For targeted scRNAseq, single CD3^+^CD45^+^DAPI^-^ cells were sorted into individual wells of a 96-well plate, snap frozen, and then thawed to lyse the cells. Cell lysates were reverse transcribed and amplified incorporating primers targeting immune phenotype genes and *TRAV* and *TRBV* gene segments (**Supplementary Table 1**). PCR amplification and barcoding were performed as described (18). Samples were pooled and sequenced using the Illumina MiSeq reagent kit v2, 500-cycles (Illumina, San Diego, CA) in the Genomics Core Facility at our center. For whole transcriptome scRNAseq, sorted CD3^+^CD45^+^DAPI^-^ cells from single-cell suspension samples derived from three patients were loaded at 17,000 cells per lane onto a 10X Genomics Controller (10X Genomics, Pleasanton, CA). scRNAseq libraries were constructed according to the manufacturer’s protocol. Pooled 3’ V(D)J and 5’ GEX libraries were sequenced on a NovaSEQ SP100 flowcell (Illumina) to obtain 5,000 reads/cell and 20,000 reads/cell depth, respectively.

### Bioinformatic analysis

TRB-CDR3 repertoire analyses were conducted using the LymphoSeq R package (http://bioconductor.org/packages/LymphoSeq). Files containing unique TRB variable region nucleotide sequences with associated CDR3 amino acid sequence, read count, frequency, and VDJ gene segment names were exported from the Adaptive Biotechnologies ImmunoSEQ analyzer v2. Non-productive sequences were removed. Normalized Shannon entropy (the frequencies of all productive sequences divided by the logarithm of the total number of unique productive sequences) value was inverted (1 - normalized entropy) to produce clonality. The similarity score was defined as the number of shared unique sequences between two samples as a fraction of the total number of unique sequences in the two samples. The TRB CDR3 amino acid sequences in each repertoire were compared with the VDJdb database (19) of annotated TRB sequences to identify sequences that have previously been associated with a T-cell response to a specific microbial or tissue antigen or a specific pathological condition. To track T cell clone abundance across different tissues and time, alluvial plots featuring the 120 most frequent TRB-CDR3 sequences in the NAT, tumor, and PBMC repertoires of a patient were generated. The GLIPH2 R package was used to identify “specificity groups” comprising closely related sequences with predicted similar or identical antigenic specificity within the TRB-CDR3 sequence repertoires (20). T cell packing plot were created using igraph package. Outer circles represent GLIPH2 specificity groups comprising TRB-CDR3 amino acid sequences with predicted similar or identical antigenic specificity; inner circles represent unique TRB-CDR3 amino acid sequences. For targeted scRNAseq libraries, outputs were de-multiplexed using customized R scripts and the CDR3 regions with associated V(D)J region information were extracted with MiXCR alignment (21). Targeted phenotype analysis was performed with the Seurat package (22) using the FindAllMarkers function to find differentially expressed genes from each identity cluster against the remaining cells, with min.pct = 0.25, log fc.threshold = 0.25. Pathway analysis was conducted using the clusterProfiler (23) and ReactomePA R packages (24). The 10X Chromium scRNAseq outputs were de-multiplexed and mapped to the human reference genome (hg19, GRCh38) through the Cell Ranger V4.0 bioinformatics pipeline (10X Genomics). Correction for batch effects was performed as described (25). Dimension reduction and clustering of the transcriptome-wide scRNAseq data were conducted using the PAGA algorithm incorporated in the Monocle3 package (26–28). To extract marker genes for each cell module, a “top markers” command was executed on a single-cell object. The cell classification was performed with the Garnett R package (29) based on marker genes derived from bulk RNA-seq datasets (30) that have a false discovery rate < 0.001, as well as gene markers compiled from multiple RNA sequencing references (**Supplementary Table 2**).

## Results

### mIHC analysis of renal tumors reveals heterogeneity of intra-tumoral and inter-patient T-cell enrichment

**Figure 1:**
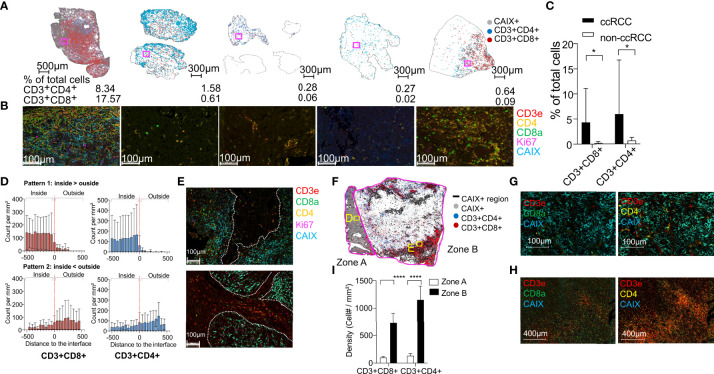
Renal tumors with diverse histologies have different densities of T-cell infiltration and heterogeneous spatial distribution of T cells in ccRCC. **(A)** Distribution of CD4^+^ (blue) and CD8^+^ (red) T-cells in whole tissue sections from the tumor cases with identification of the CAIX^+^ regions (gray) in each tumor. Tumor types include clear cell (cc), chromophobe, and papillary subtypes, one patient with an oncocytoma, and one patient with a sarcoma. The percentage of CD3^+^CD4^+^ and CD3^+^CD8^+^ T cells of the total cell count in each tumor section is indicated at the bottom. **(B)** Representative mIHC images of tumor-infiltrating T cells in the area enclosed within the magenta box in each tissue section. **(C)** Quantification of CD3^+^CD4^+^ and CD3^+^CD8^+^ T cells in the corresponding whole tumor sections from ccRCC (n=10) and non-ccRCC tumors (n=4). Error bars indicate standard deviation. *P < 0.05, t-test. **(D)** Two distinct patterns of CD3^+^CD8^+^ (left column, red) and CD3^+^CD4^+^ (right column, blue) T-cell density within 500µm of the tumor border in eight cases of ccRCC. The red dashed line indicates the tumor border. Negative and positive distances indicate regions inside and outside the tumor, respectively. Top row: T-cell density higher inside than outside tumor (n=5); bottom row: T-cell density higher outside than inside tumor (n=3). **(E)** mIHC images of T-cell distribution in one representative tumor from each group. The white dotted lines mark the CAIX^+^ tumor borders. **(F)** Distribution of CD3^+^CD4^+^ (blue) and CD3^+^CD8^+^ (red) T cells, and CAIX^+^ tumor cells across an entire tumor section from ccRCC patient 13. Discrete CAIX^+^ (zone A) and CAIX^-^ (zone B) regions within the tumor are outlined by magenta lines. **(G, H)** mIHC images of CD3^+^CD4^+^ and CD3^+^CD8^+^ T cells in the regions enclosed within the yellow squares in panel C **(I)** Mean CD3^+^CD8^+^ and CD3^+^CD4^+^ T-cell density in eight representative CAIX^+^ (zone A) and 8 representative CAIX^-^ (zone B) square regions (100 µm edge) in a ccRCC tumor from patient 13. Error bars indicate standard deviation. *P ≤ 0.05, ****P ≤ 0.0001, t-test.

To study the intensity and spatial heterogeneity of T-cell infiltration of renal tumors, we collected renal tumor, NAT, and PBMC from 14 consecutive nephrectomy cases. Pathologic analysis demonstrated 10 ccRCC tumors, and four non-clear cell renal tumors (chromophobe RCC, papillary RCC, oncocytoma, and sarcoma) (**Table 1**). mIHC with CD3, CD4 and CD8 T-cell markers revealed distinct patterns of T-cell infiltration in the different tumor subtypes (**Figures 1A, B**). We observed significantly higher CD3^+^CD4^+^ (> 8.6-fold) and CD3^+^CD8^+^ (> 22.2-fold) T-cell infiltration into ccRCC compared to the non-ccRCC tumors (**Figure 1C**), consistent with previous reports (31, 32). We used the ccRCC marker CAIX to distinguish regions of viable tumor from necrotic tumor and stroma to characterize T-cell distribution with respect to viable tumor domains. Eight of 10 ccRCC cases were stained strongly positive for CAIX. Five of eight CAIX^+^ tumors had increased CD4^+^ and CD8^+^ T-cell infiltration into the CAIX^+^ areas (**Figures 1D, E** top panels), whereas in three tumors, the opposite pattern was observed, with CD4^+^ and CD8^+^ T cells being mostly excluded from the CAIX^+^ tumor regions (**Figures 1D, E** bottom panels). Two of the five T-cell infiltrated CAIX^+^ ccRCC tumors showed significant heterogeneity of intra-tumoral T-cell infiltration, with frequent CD8^+^ and CD4^+^ cells observed in some tumor regions with discrete CAIX expression but far fewer such cells in other contiguous CAIX-expressing areas (**Figures 1F–I**). These data are consistent with a recent related study (9), and indicate that immune infiltration in ccRCC at the time of diagnosis is characterized by marked spatial heterogeneity in T cell distribution and density.

### TCR repertoire profiling reveals clonal expansion as a phenotype of tumor-associated T cells

**Figure 2:**
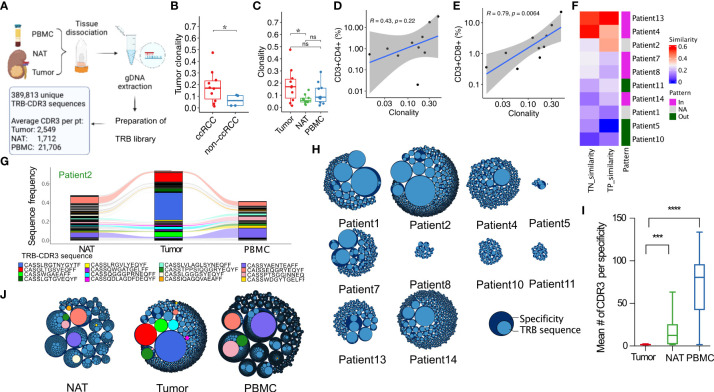
T-cell receptor β-chain (*TRB*) repertoire sequencing reveals RCC-associated *TRB* sequences that are expanded in RCC tumors. **(A)** Workflow and output of *TRB* repertoire sequencing. **(B)** Clonality (see methods for definition) of TRB repertoires in ccRCC and non-ccRCC tumors. **(C)** Clonality of TRB repertoires in tumor, NAT, and PBMC from ccRCC. **(D)** Correlation between tissue CD3^+^CD4^+^ or **(E)** CD3^+^CD8^+^ infiltration and TRB repertoire clonality in 10 ccRCC tumors. **(F)** Repertoire similarity between pairs of tissues in the indicated ccRCC patients. TN: tumor and NAT; TP: tumor and PBMC. Pattern annotation: the T-cell infiltration pattern defined in Figure 2A. Magenta: T cells infiltrated into CAIX^+^ viable tumor area more than CAIX^-^ area; green: T cells infiltrated into CAIX^-^ area more than CAIX^+^ area; gray: ccRCC tumors with weak CAIX staining. Similarity score was defined as the number of shared unique sequences between two samples as a fraction of the total number of unique sequences in the two samples. **(G)** Alluvial plot of the 120 most frequent TRB-CDR3 sequences in the NAT, tumor, and PBMC of patient 2. Rivulets identify TRB-CDR3 sequences that are shared between two or three tissues. **(H)** Packing plot representations of GLIPH2 analysis of TRB-CDR3 repertoire composition of 10 ccRCC tumors. Dark blue outer circles: GLIPH2 specificity groups comprising TRB-CDR3 amino acid sequences with predicted similar or identical antigenic specificity; light blue inner circles: unique TRB-CDR3 amino acid sequences. The diameter of the light blue inner circles is proportional to the frequency of the corresponding TRB-CDR3 sequence in the repertoire of that sample. The overall size of each repertoire is proportional to the total number of defined specificity groups in each sample. **(I)** Mean number of unique TRB-CDR3 sequences in GLIPH2-defined specificity groups in tumor, NAT, and PBMC from patients with ccRCC. **(J)** GLIPH2-defined repertoire composition in tissues from patient 2. Colored circles correspond to the TRB-CDR3 amino acid sequences in panel G. *P ≤ 0.05, ***P ≤ 0.001, ****P ≤ 0.0001, ns not significant P > 0.05, t-test.

To evaluate the TCR diversity and clonal composition of T cells infiltrating into renal tumors, we performed deep sequencing of the hypervariable CDR3 domain of the *TRB* gene using genomic DNA extracted from T cells purified from renal tumor, NAT, and PBMC from 14 subjects. In total, we identified 389,813 unique TRB-CDR3 sequences, and an average of 2,549 unique sequences (range 25-6,693) per tumor (**Figure 2A**). Analysis of the most frequently observed intra-tumoral TRB-CDR3 sequences, each serving as a marker for an expanded T-cell clone (a clonotype), demonstrated that nine out of 14 patients harbored a single T-cell clone dominating the TIL repertoire and comprising 4.6% to 24% of total TRB-CDR3 sequences (**Supplementary Figure 1A**). The TIL TCR repertoire in an additional patient (patient 1) showed a pattern characterized by several dominant clonotypes expanded at similar frequencies. The TIL TCR repertoire from the cohort of ten ccRCC tumors demonstrated significantly higher clonality than was observed in the four non-ccRCC tumors (**Figure 2B**). The T-cell clonality in ccRCC tumors was also higher than in NAT (**Figure 2C**), identifying clonal expansion as a phenotype of the tumor-associated T-cell repertoire in ccRCC. Moreover, CD8^+^ but not CD4^+^ T-cell tumor infiltration as revealed by IHC (**Figures 2D, E**) correlated significantly with clonality of the TIL repertoire, highlighting the dominance of CD8^+^ T-cell clonal expansion in the ccRCC TME.

In total, 2,759 expanded TCR clonotypes (defined as unique TRB-CDR3 amino acid sequences with a frequency > 1% in TIL repertoires) were discovered. To address the possibility that expanded clones within TIL samples represented “bystander” T cells recognizing non-tumor-associated antigens, we compared the TRB-CDR3 amino acid sequences to the VDJdb database of annotated TRB sequences (19) to identify sequences that have previously been associated with a T-cell response to a specific microbial or tissue antigen or pathological condition. Only 194 unique TRB-CDR3s (0.05%) from the 14 TIL repertoires matched CDR3s from TCRs that have previously been associated with T-cell responses to one of the 13 defined pathogens or public tumor-antigen targets (non-RCC), with individual clonal expansion no greater than 1.3% of the corresponding sample repertoire (**Supplementary Figure 1B**). Clonotypes in TIL repertoires potentially reactive with a specific non-tumor related target account for ≤ 7% of the TIL repertoire in each patient (**Supplementary Figure 1C**). TRB-CDR3s were also screened against a TCR sequence library with greater than 11.7 million unique TRB-CDR3 sequences pooled from PBMC collected from 55 control subjects without cancer as a tool to interrogate shared specificity for non-cancer-associated target antigens (33, 34). 63.9% of TIL-derived, expanded *TRB*-CDR3s with clonal frequency over 1% were patient-unique and not identifiable in this control sequence library (**Supplementary Figure 1D**). Taken together, these data exclude expanded clones specific for common pathogens as the explanation for the unique clonal architecture of RCC TIL. Instead, expanded T cell clones in ccRCC carry TCRs with primarily private specificities that are not commonly shared with healthy subjects supporting the idea that the clonally expanded T cells may be reacting against RCC-associated targets.

The TCR repertoires of TIL from each tumor had a subset of sequences in common with the repertoires isolated from autologous NAT or PBMC. To quantify the sequence overlap, a similarity score was calculated representing the number of shared unique sequences between two samples normalized to the total number of unique sequences. Most of the subjects with higher sequence similarity between TIL and NAT or PBMC repertoires had “hot” tumors with dense T-cell infiltration in CAIX^+^ tumor regions revealed by IHC, whereas the subjects with low repertoire similarity between the three tissues had “cold” tumors characterized by CAIX^+^ areas with few T cells (**Figure 2F**). The TRB-CDR3 sequences in TIL repertoires were more frequently shared with PBMC than NAT (**Supplementary Figure 2A**). In five ccRCC patients, the TIL repertoire also contained clonotypes expanded with a frequency ≥ 1% that were not identified in either PBMC or NAT. For example, in subject 2, three clonotypes that were detected only in the RCC tumor comprised ~60% of the entire TIL repertoire (blue TRB-CDR3 sequence: CASSLRGTNYGYTF; red TRB-CDR3 sequence: CASGLTGSVEQFF; green TRB-CDR3 sequence: CASSWGAEAFF) (**Figure 2G**). A follow-up PBMC sample was obtained from subject 7 one year after initial nephrectomy. TCR repertoire analysis revealed that multiple expanded TRB-CDR3 clonotypes observed in tumor, NAT, and PBMC at frequencies up to 3% at the time of surgery were maintained in peripheral blood at high frequency (up to 3.8%) one year later (**Supplementary Figure 2B**). Thus, intensely infiltrated tumors exhibited higher TRB-CDR3 amino acid sequence similarity between the tumor, NAT and PBMC. Furthermore, this TIL-derived TCR repertoire of expanded TRB-CDR3 sequences was a variable and possibly stable composite of sequences shared on T cells trafficking in blood or normal tissues and sequences unique to the TME.

We speculated that among expanded TRB-CDR3 sequences found in TIL repertoires, unique TRB-CDR3s may share specificity targeting the same tumor-associated antigens. To explore this possibility, we used the GLIPH2 algorithm (20) to group RCC TIL-derived TRB-CDR3s by their predicted specificity based on CDR3 sequence motifs (**Figure 2H**). When compared with peripheral blood and NAT-derived repertoires, GLIPH2 specificity clustering of TIL repertoires contained significantly fewer unique TRB-CDR3 sequences per specificity group (**Figures 2I, J**), and in general these were comprised of a single or at most 2-3 unique sequences. Thus, GLIPH2 analysis suggested either selectivity or exclusion of T cells entering the TME amongst a larger pool of cells with shared antigen specificity circulating in the peripheral blood and capable of trafficking into NAT.

To further explore shared T-cell specificity for common antigen targets between RCC tumors, GLIPH2 analysis was applied to pooled TIL repertoires selected from subjects with an *HLA* allele in common. GLIPH2 clustering of pooled TRB-CDR3 sequences from the most frequent *HLA-A* (HLA-A*02:01, 6 tumors) or -*B* alleles (HLA-B*35:01, three tumors) among the 10 ccRCC tumors revealed only rare *TRB* specificity grouping with sequences from more than one patient (**Supplementary Figures 2C, D**) and no clusters with sequences from more than two patients. This outcome is consistent with RCC TIL repertoires being dominated by private antigen specificities.

### Targeted scRNAseq reveals expanded T-cell clones in RCC tumors that express both Ki67 and CTLA4

**Figure 3:**
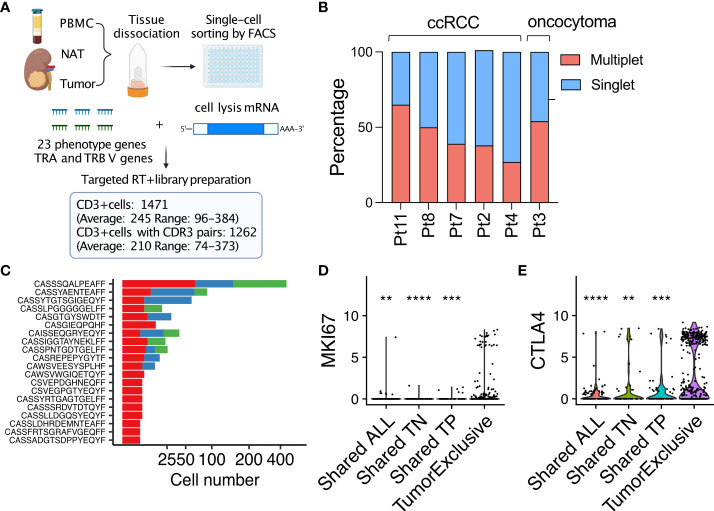
Targeted scRNAseq reveals that tumor-exclusive T-cell clonotypes are associated with distinct phenotypes compared to NAT and PBMC. **(A)** Workflow and output of targeted single T-cell RNAseq. **(B)** Dichotomized frequency distribution of tumor-infiltrating T cells in 5 ccRCC cases and an oncocytoma. Multiplet, a *TRA/TRB-*CDR3 sequence pair detected more than once in a tumor sample; singlet, a *TRA/TRB-*CDR3 sequence pair detected only once in a sample. **(C)** The 20 most frequent multiplet *TRA/TRB-*CDR3 sequence pairs across all 5 ccRCC cases. **(D)** Violin plot of *MKI67* or **(E)** *CTLA4* expression in tumor-infiltrating T cells with different patterns of tissue distribution. In violin plots, the log_10_ gene expression is plotted for each single T cell (dot event). **P ≤ 0.01, ***P ≤ 0.001, ****P ≤ 0.0001, t-test.

To investigate the phenotypes and functional capacity of clonally expanded T cells within the TIL repertoires from renal tumors, we implemented a targeted scRNAseq platform with primer pairs designed to amplify the CDR3 regions of the *TRA* and *TRB* genes as well as 23 genes defining T-cell phenotypes (**Supplementary Table 1**). A total of 1,471 single CD3**^+^** cells were flow-sorted from tumor, NAT, or PBMC samples collected from five patients with ccRCC and one patient with an oncocytoma (**Figure 3A**). A range of 55-151 single CD3^+^ cells were characterized from each of the six tumor samples. Compared with NAT and PBMC, CD3^+^ T cells from TIL demonstrated robust expression of cytokine genes *IL2*, *IL12A*, *IL10*, and *IL13;* cytotoxicity genes *IFNG, GZMB, PRF1, TNFA*, and *TGFB*; the proliferation marker *MKI67*, the transcription factors *GATA3 and TBET;* and the activation-induced/exhaustion genes *CTLA4* and *EOMES*, but low-level expression of the transcription factors *RORC* and *RUNX1* (**Supplementary Figure 3A**), suggesting that the frequency of Th17 and CD4 T cells is lower in RCC tumor than in the two the normal tissues.

Despite the limited sampling depth of this targeted scRNAseq platform when compared with our high-throughput, DNA-based *TRB* repertoire analyses of the same samples, an average of 45.5% (range, 27.4 – 65.3% across six tumors) of the TRA/TRB-CDR3 sequence pairs identified in tumor-infiltrating T cells were observed in two or more cells, representing “expanded” clones (**Figure 3B**). Moreover, expression of functional markers (*IFNG, GZMB and PRF1)*, effector transcription factors *(TBET and RUNX1)* and activation/exhaustion markers *(CTLA4 and EOMES)* were upregulated in expanded T-cell clonotypes (defined by TRA/TRB-CDR3 sequence pairs) that were detected multiple times in tumors while T cells that were observed only once were characterized by higher levels of *IL2* and *RORC* expression (**Supplementary Figure 3B**). These observations confirm and extend recent single-cell analyses of RCC-derived TIL that indicate that clonally expanded T cells are enriched for effector T cells with an exhausted phenotype (12).

The most frequently observed expanded TRA/TRB-CDR3 sequence pairs (clonotypes) were detected in cells from two or more tissues (**Figure 3C**). However, many expanded TRA/TRB-CDR3 sequence pairs were only observed in TIL (at up to 14 instances per tumor sample). Compared with expanded T-cell clonotypes that were found in two or more tissues, expanded T-cell clones that were exclusively found in tumor demonstrated elevated expression of the proliferation marker *MKI67* (**Figure 3D**) and the antigen-induced marker *CTLA4* (**Figure 3E**). In all five ccRCC tumors analyzed by targeted scRNAseq, expression of *MKI67* was primarily observed in T-cell clones found exclusively in tumor for every case (**Supplementary Figure 3C**). Thus, despite high expression of *CTLA4*, indicating late stage of antigen-experienced differentiation, a subset of tumor-associated expanded T cell clones remained capable of continued proliferation and expansion within the TME.

### High-throughput, whole transcriptome scRNAseq phenotype analysis of expanded T-cell clones in RCC tumors

**Figure 4:**
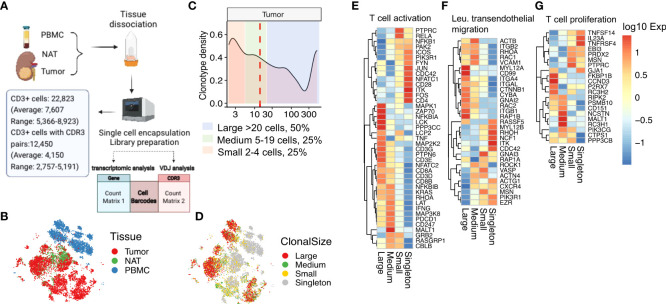
Expanded tumor-infiltrating T cells upregulate TCR activation and migration pathways. **(A)** Workflow and output of 10X Genomics targeted 3’ V**(D)**J sequencing and 5’ whole-transcriptomic profiling. **(B)** tSNE clustering of gene expression profiles of single T cells derived from tumor, NAT, and PBMC. **(C)** Plot of clonotype density, defined as the number of unique TRA/TRB sequence pairs as a percentage of the total number of unique TRA/TRB sequence pairs, as a function of clonal size. Median clonal size is marked by the red dashed line. Areas shaded in different colors represent ranges of different clonal size. Large clones, >20 cells (light blue); medium clones, 5-20 cells (light green); small clones, 2-4 cells (light pink). **(D)** tSNE clustering of the single T cells in **(B)** colored according to their respective clonal sizes. Singletons refer to TRA/TRB sequence pairs observed only once. **(E)** Heatmap of expression in tumor-infiltrating T cells of genes in the T-cell activation gene set (GO:0042110), **(F)** the leukocyte transendothelial migration gene set (hsa04670), and **(G)** the T-cell proliferation gene set (GO: 0042098).

We transitioned our single cell analyses to the 10X Genomics microfluidic partitioning platform to gain higher throughput and deeper sampling of the T cell transcriptomes in tumor, NAT, and PBMC samples than achieved by our targeted scRNAseq platform. Using this platform, we profiled a total of 22,823 CD3**^+^** T cells (average: 7,607; range: 5,366-8,923) isolated from tissues from three additional subjects (**Figure 4A**) representing a 28-fold increase in throughput per patient, on average, compared with our targeted scRNAseq analyses. We identified 12,450 cells associated with TRA/TRB-CDR3 pairs (average: 4,150; range: 2,757-5,191), an 18-fold per patient increase over our targeted scRNAseq analyses (**Supplementary Figure 4A**). The linear correlation of *TRB* clonal frequency by whole transcriptome scRNAseq compared with bulk DNA template-based TCR repertoire profiling suggested that the T-cell composition could be accurately sampled from different tissues by whole transcriptome scRNAseq of paired TRA/TRB recombination regions (**Supplementary Figures 4B, C**). It was also possible to accurately associate the expression phenotype and tissue distribution for a given TRB-CDR3 amino acid sequence detected in bulk DNA template-based TCR repertoire sequencing by matching the TRB-CDR3 sequences. Moreover, we compared the number of highly abundant clones that were identified by bulk DNA template-based TCR repertoire profiling with the two scRNAseq platforms. The whole transcriptome scRNAseq platform consistently detected more highly abundant clones than targeted scRNAseq, and the latter platform demonstrated more interpatient variability (**Suppementary Figure 4D**).

Dimensionality reduction and t-distributed stochastic neighbor embedding (tSNE) clustering analysis of the CD3^+^ *TRA/TRB-* expressing T cells from tumor, NAT, and PBMC revealed distinct clusters containing cells from tumor compared with cells from PBMC, with cells from NAT located primarily within or along the margins of the tumor cluster (**Figure 4B**). Consistent with our observations from targeted scRNAseq, the whole transcriptome scRNAseq data showed that the tumor-infiltrating T cells are phenotypically distinct in comparison to T cells isolated from NAT or peripheral blood. TILs were enriched for CD8^+^ cells expressing high levels of effector/cytokine (*GZMB, IFNG, IL2 and IL12A*) and repetitive antigen encounter/exhaustion (*PDCD1, CTLA4*) genes (**Supplementary Figure 5A**).

### The expansion size of clonal T cells in RCC tumors correlates with distinct phenotypes

From the whole transcriptome scRNAseq data analyzed for TRA/TRB-CDR3 pairs, we detected 53.5% CD3^+^ cells with expanded TCR clonotypes (i.e., TRA/TRB-CDR3 pair detected more than one time). We defined large, medium, and small clonotypes (large clones, >20 cells, 50% of total expanded cells; medium clones, 5-20 cells, 25% of total expanded cells; small clones, 2-4 cells with identical TRA/TRB CDR3 pairs, 25% of total expanded cells). Singletons refer to the T-cell clonotypes with a TRA/TRB-CDR3 pair observed only one time (**Figures 4C, D**). The tumor-infiltrating T cells with a larger clonal size were enriched for *CD8* expression, whereas *CD4* expression was associated with singletons and small clonal size. Regarding the top differentially expressed marker genes, the naïve markers *IL-7R* and *LTB* were primarily expressed on singletons whereas the chemokine *CCL5*, the cytotoxicity regulator *CST7*, and the cytotoxicity genes *NKG7*, *GZMB* and *GZMH* were more highly expressed on expanded T cells (**Supplementary Figure 5B**).

This observation led us to examine pathways that were differentially expressed in these cells. Indeed, gene expression profiling for markers of T-cell activation, leukocyte transendothelial migration, and T-cell proliferation revealed that the size-based segregation of expanded TIL clonotypes was associated with unique expression profiles (**Figures 4E–G**). Large clones had upregulated canonical T-cell activation pathway genes including *MAPK1, ZAP70* and *LCK* and had decreased expression of the *JUN/FOS* axis, *PIK3R1, ITK, FYN*, and the costimulatory molecule *CD28* (**Figure 4E**) consistent with antigen driven expansion. The large T-cell clones had upregulated expression of the small GTPases *RHOA, RAP1B, RAC1* and *RAC2* in the T-cell transendothelial migration pathway, consistent with a “go” signal for enhanced migration (35) (**Figure 4F**). In contrast, the singletons and small clones had increased *RHOH* expression which has been shown to inhibit chemokine-induced T-cell migration (36). The large clonotypes expressed proliferation genes including *FKBP1B, CCND3, P2RX7*, while the singletons expressed proliferation cytokines *IL23A, TNFRSF4* and *TNFRSF14* (**Figure 4G**). Overall, this result suggested that the magnitude of clonal expansion associates with T-cell subtypes with different phenotypes and functional capacity. The T-cell clonotypes with a larger clonal size exhibit a CD8 cytotoxic phenotype, while the clonotypes with smaller clonal size remain naïve or of CD4 origin. Distinct T-cell activation and migration pathways are associated with cytotoxic or naïve T cells with different clone size.

### The tissue distribution of clonal T cells in RCC tumor reveals an effector T-cell population shared by normal tissues and a tumor-restricted, exhausted memory population

**Figure 5:**
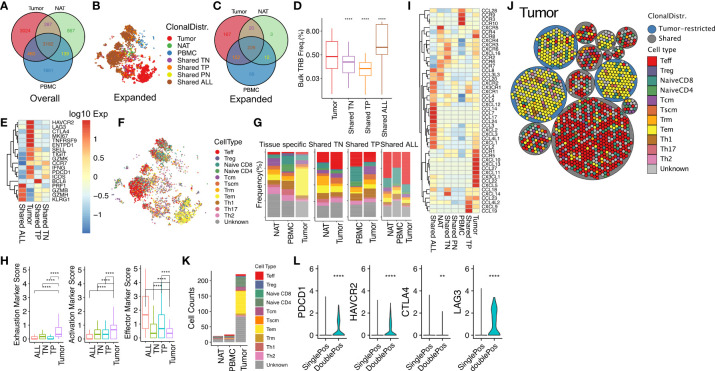
Effector T cells found in multiple tissues and exhausted memory T cells found only in RCC tumor express unique patterns of chemotaxis markers as clues for distinct tissue migration. **(A)** Venn diagram of the numbers of unique TRA/TRB sequence pairs in RCC tumor, NAT, and PBMC. **(B)** tSNE plot of expanded T-cell clonotypes with different tissue distribution: tissue specific (tumor, NAT, PBMC); shared by two tissues (TN, TP, TN), or shared by all tissues (ALL). Similar to the targeted scRNAseq, clonotype was defined as a TRA/TRB-CDR3 pair identified from the same T cell. **(C)** Venn diagram of expanded clonotype counts from tumor, NAT and PBMC. The numbers indicate the unique TRA-TRB sequence pairs detected in each compartment. **(D)** Frequency of tumor-infiltrating T-cell clonotypes with different tissue distributions (Bulk TRB Frequency was derived from TRB repertoire sequencing with matched TRB-CDR3 sequence detected in scRNAseq). **(E)** Expression of marker genes in expanded tumor-infiltrating T cells categorized by tissue distribution. **(F)** Phenotypic classification of the expanded TRA/TRB sequence pairs in panel B **(G)** Frequency distribution of expanded clonotypes according to phenotype and tissue distribution. **(H)** Mean expression of gene sets associated with exhaustion (*ICOS*, *TIM3*, *CTLA4*, *PD1*, *LAG3*, *TIGIT)*, activation (*TNFSR9*, *CD69*, *CD25)*, and effector function (*GZMB*, *PRF1*, *IFNG)* in expanded TIL. **(I)** Expression of genes in the KEGG chemokine signaling pathway (hsa04062) in T cells infiltrating into tumor, NAT and PBMC with different tissue distribution. **(J)** Packing plot of TIL clonotypes with frequency >= 1% in tumor classified by phenotype and tissue distribution. Large circles: cells with same clonotype (*TRA/TRB*-CDR3 pair); small circles: individual cells. Blue clonotypes were exclusively found in tumor, and grey circles were tumor-derived clonotypes shared by one or two normal tissues. **(K)** Phenotypic classification of *CD4* ^+^ *CD8^+^* T cells in Tumor, NAT, and PBMC. **(L)** Relative expression of *PDCD1, HAVCR2, CTLA4* and *LAG3* in *CD4* or *CD8* single positive or double positive cells. **P ≤ 0.01, ****P ≤ 0.0001, ns not significant P > 0.05, t-test.

We categorized all unique clonotypes (unique *TRB/TRA*-CDR3 pair sequences) based on their tissue association into seven possible tissue distribution patterns (tissue specific: tumor, NAT, PBMC; shared by two tissues: tumor-NAT (TN), tumor-PBMC (TP), PBMC-NAT (PN); or shared by all tissues: ALL). We then similarly segregated the expanded clonotypes (**Figures 5A–C**). Among tumor-associated clonotypes (tumor, TN, TP, ALL), 70% of the 565 tumor-associated, expanded T-cell clonotypes were shared with one or more other tissues. Clonotypes shared by all tissues (226/565, 40%) comprised the most frequently observed tissue distribution pattern. The clonotype size differed among tumor-associated TCRs with different patterns of tissue distribution. The TCRs shared by all tissues had the highest average clonal size (**Figure 5D**) reflecting the phenotype of large clonotypes described above. Effector genes such as *GZMB, GZMH* and *KLRG1* were the marker genes in clonotypes shared by all tissues, while the clonotypes present only in tumor showed upregulated T-cell exhaustion genes (*HAVCR2, LAG3, CTLA4, TIGIT, PDCD1, and ICOS)* and activation genes (*SELL* and *TNFRSF9*) (**Figure 5E**).

We then correlated the tissue distribution pattern of expanded T-cell clonotypes with functional T-cell subtype classification (**Figures 5F, G**). Tumor-restricted clonotypes were enriched for a T effector memory (T_em_) population (yellow) compared with expanded clonotypes present only in NAT or PBMC. In contrast, T cells with clonotypes shared by all tissues were highly enriched for a T effector (T_eff_) population (red). We observed significantly higher levels of exhaustion (*ICOS, TIM3, CTLA4, PD1, LAG3, TIGIT*) and activation (*TNFSR9, CD69, CD25*) markers for clonotypes restricted to the tumor (**Figure 5H**). In addition, the tumor-restricted clonotypes expressed lower effector markers (*GZMB, PRF1, IFNG*) compared with clonotypes shared with other tissues, indicating possible antigen-experience and exhaustion of TCR clonotypes in the TME. Most of the singleton cells were central memory T cell (T_cm_), stem-cell like memory T cell (T_scm_), and CD4^+^ T cells comprised of Th1, Th2, Th17 and regulatory T cells (T_reg_). In general, CD4^+^ T cells tended to have lower clonal size or cell counts, regardless of their phenotype.

Collectively, these data point to the presence of two distinct populations of expanded T cells in tumor: 1), a T_eff_ population expressing a “go” signal and interstitial migration genes with large clonal size refreshed from T cells in the circulation, and 2), a T_em_ population with the TCRs only present in tumor, expressing markers of antigen experienced markers and exhaustion genes, and high expression of *RHOH* as an inhibitory signal for chemokine mediated migration.

We speculated that chemokine signaling plays an important role in controlling T-cell subtype distribution in the TME. T-cell clonotypes restricted to tumor expressed a characteristic set of chemokines and chemokine receptors including *CCR1*, *CCR5* and *CXCL5*. In contrast, the T-cell clonotypes shared by all tissues expressed a unique group of chemokine ligands and receptors including the pro-inflammatory chemokine receptor *CX3CR1* (**Figure 5I**). This result suggests the presence of tumor-specific homing cues that preferentially recruit T cells with a pro-inflammatory phenotype into the TME. When analyzed by T-cell subtypes, chemokine ligand and expression profiles varied widely. For example, the expression of *CCR4* was upregulated in Th2 cells, *CCR8* was upregulated in Treg cells, and *CXCL16* was upregulated in naïve CD4^+^ T cells, suggesting the presence of cell type-specific homing cues (**Supplementary Figure 5C**).

### Expanded TCR clonotypes in RCC tumors include CD4^+^CD8^+^ double positive T cells

We observed that T-cell clonotypes in TIL were often composed of a range of phenotypic subpopulations, suggesting phenotypic differentiation within the TME (**Figure 5J**). A subset of clonotypes were composed of individual cells mapping to both the CD4 and CD8 lineages. To exclude possible doublets, we filtered out cells with more than one pair of TRA/TRB-CDR3s detected. Globally, we detected 266 CD4^+^CD8^+^ double positive T cells out of 12,039 total cells (2.2%) analyzed with whole transcriptome scRNAseq platform, and 221 (83%) of them were from tumor, representing 4.5% of the 4,844 tumor-derived T cells. Interestingly, Interestingly, all TCR clonotypes that included CD4^+^CD8^+^ double positive T cells were also expressed by additional single positive cells (**Supplementary Figure 6A**). Among 56 tumor-infiltrating T cell clonotypes that contained double positive cells, 26 were restricted to tumor, 20 were shared by all tissues and 10 were shared with NAT. Tumor-derived double positive T cells had a higher rate of clonal expansion (89.6%) compared with overall TIL (70.6%). Further analysis identified *CD4* and *CD8* co-expression by 10 out of 52 top expanded tumor-derived clonotypes with clonal frequency over 1%. Tumor-associated double positive expanded clonotypes frequently displayed a T_em_ phenotype and expressed elevated exhaustion markers *PDCD1, TIM3, LAG3* and *CTLA4* (**Figures 5K, L**) similar to tumor-restricted CD8^+^ expanded clonotypes. The top differentially expressed gene pathways in tumor-infiltrating double positive cells versus single co-receptor expressing cells from shared clonotypes were related to MHC antigen presentation, CD28 stimulation and TCR signaling, interferon signaling, and PD-1 signaling (**Supplementary Figures 6B, C**). Among all 56 unique double positive T cell associated clonotypes, only one TCR sequence had identity with a defined antigen specificity (TRB: CASSPGQGGGYTF; TRA: CAVHNTDKLIF; antigen: M. Tuberculosis) (20, 37). Taken together, our data indicate the phenotype of CD4^+^CD8^+^ double positive T cell clonotypes is consistent with target antigen recognition in the TME (38).

### Ki67^+^ TCR clonotypes co-localize with CAIX^+^ viable tumor domains *in situ*

**Figure 6:**
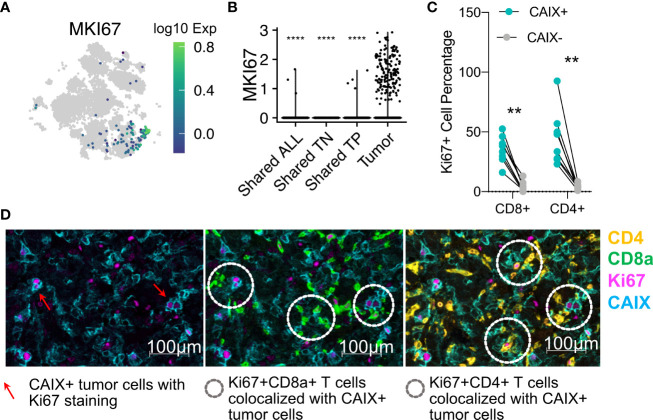
Tumor-restricted T cells express the *MKI67* marker of proliferation, and directly contact CAIX^+^ tumor cells *in situ.* **(A)** tSNE projection of all T cells from 10X genomics analysis colored according to *MKI67* gene expression. **(B)** *MKI67* expression in TILs with different tissue distributions. The t-test comparisons were relative to tumor group (tumor-specific T cells). **(C)** Ki67^+^CD3^+^CD4^+^ or CD8^+^ as a percentage of the total numbers of CD3^+^CD4^+^ or CD8^+^ as determined by mIHC in eight ccRCC whole tumor scans. **(D)** Representative (Patient 10) Ki67 protein expression on CD3^+^CD8^+^ or CD4^+^ T cells infiltrating into Ki67-expressing CAIX^+^ tumor. Red arrows indicate the proliferating CAIX^+^ tumor cells in panel 1. White doted circles in panel 2 and three indicate the interactions of proliferating lymphocytes with proliferating tumor cells. **p ≤ 0.01, ****p ≤ 0.0001, t-test.

Our analyses of both the targeted and high-throughput whole transcriptome scRNAseq data sets demonstrated that the proliferation marker *MKI67* was primarily expressed on expanded, tumor-restricted TCR clonotypes (**Figure 6A**). The *MKI67* marker was associated with clonotypes with a T_em_ phenotype (**Figures 5F**, **6B**). These data suggest that T cell proliferation was occurring within the TME. To evaluate the *in situ* localization of T cells positive for Ki67, we performed mIHC on eight CAIX^+^ ccRCC tumors. This analysis revealed that proliferating Ki67^+^CD3^+^CD8^+^ and Ki67^+^CD3^+^CD4^+^ T cells were in close proximity to CAIX-expressing tumor cells (**Figures 6C, D**). Taken together, these data suggest direct interactions of proliferating, expanded T_em_ cells with tumor cells within the TME.

### Analysis of shared TCR clonotypes among RCC tumors identifies rare putative public TCRs

**Figure 7:**
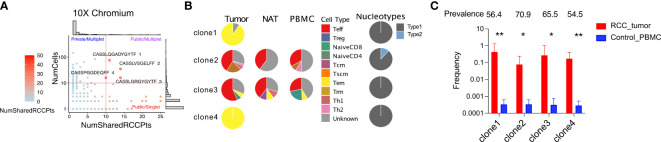
scRNAseq reveals expanded *TRB*-CDR3 sequences shared by multiple RCC patients. **(A)** For a given TRA/TRB pair, the number of RCC patients in whom that sequence pair observed over the number of cells detected were plotted from whole transcriptome sequencing. **(B)** The phenotype of the four most frequent public and expanded TRB-CDR3 amino acid sequences. Nucleotype was defined as unique nucleotide sequences encoding the same amino acid sequence. **(C)** The frequency of the four public TRB-CDR3 sequences in RCC tumors and control individuals. The frequency is calculated from DNA-sequencing of TCR repertoire from corresponding samples. Prevalence is calculated as the percentage of 55 control PBMC donors who carry a particular TRB-CDR3. Statistics: t-test, *P ≤ 0.05, **P ≤ 0.01, ****P ≤ 0.0001.

We have demonstrated that the TCR repertoire isolated from RCC tumors is primarily composed of private sequences unique to the individual tumor. To screen for rare TCR clonotypes that may be shared across multiple tumors, we compiled a TCR repertoire database including the 14 tumors analyzed in this report pooled with publicly available TCR repertoire datasets representing a total of 53 RCC tumors (16, 39, 40). We then compared all expanded TRB-CDR3 sequences identified from our targeted scRNAseq as well as our high-throughput whole transcriptome scRNAseq analyses against this RCC tumor-derived TCR repertoire database. As seen with the repertoire comparison to normal donors, the majority of TCR clonotypes identified were private to individual tumors (65.8% of the TRB-CDR3 sequences). However, we also identified four TRB-CDR3s with unique TRA pairings that were detected in over 10 cells/tumor and shared by 10 to 14 RCC tumors representing the best examples of putative “public” TCR clonotype sequences (**Figure 7A**). Two of the four TCRs (TRB-CDR3 sequences CASSLQGADYGYTF and CASSPGGDEQFF, both from patient 14) were only found in the tumor tissue with clonotype-bearing T cells adopting a uniform T_em_ phenotype and nucleotype (unique DNA sequences) across all cells (**Figure 7B**). Two additional TCRs (TRB-CDR3 sequences CASSLVSGELFF, patient 13, and CASSLGRGYGYTF, patient 14) were detected in all three tissues. Single-cell phenotypes showed heterogeneity but were dominated by a T_eff_ subpopulation in all tissues. Interestingly, the CASSLVSGELFF CDR3 protein sequence originated from two distinct nucleotide sequences, each appearing in more than one cell, suggesting the existence two precursor T-cell clones responding to a common selective pressure that drove convergent expansion of T cells with different clonal origin. Based on DNA-template repertoire analysis, the TRB-CDR3 sequences for these TCRs had a high prevalence among normal donors (56-71% of 55 normal donors) but showed preferential clonal expansion in RCC patients. The average clonal frequency was 1000-fold greater in RCC tumor than normal donors (**Figure 7C**). These data reveal the preferential clonal expansion of TCRs frequently present in healthy individuals that may be responding to RCC antigen encounter.

## Discussion

Recent single-cell sequencing analyses have drawn attention to the clonally expanded CD8^+^ T cells present in ccRCC tumors at the time of diagnosis for their association with ICI response (13, 41–43). In this report we have extended these published studies by defining the clonal architecture and phenotypes of RCC TIL, focusing particularly on the phenotype of clonally expanded T cells in the tumor microenvironment. The TCR repertoires of ccRCC TIL in our cohort were characterized by expanded CD8^+^ clones that comprised up to 24% of the entire TIL population. Although we did detect “bystander” T-cells likely specific for viral or other non-tumor antigens in every TIL repertoire, as others have reported (11, 26), the large clonal expansions we observed were mainly composed of private TCR specificities that could not readily be accounted for by a bystander response. By comparing the TCR repertoire present in ccRCC tumor with autologous NAT and PBMC using two complementary scRNAseq approaches, we identified two dominant phenotypes in the expanded clones in the ccRCC microenvironment: 1) T-cell clonotypes defined by their TRB-CDR3 sequences that were found only in tumor, and 2) T-cell clonotypes shared by tumor with PBMC and/or NAT. While all ccRCC tumors analyzed had expanded T-cell clonotypes shared with PBMC, five of 10 tumors were also found to have tumor-restricted clonotypes that represented over 1% of total clonotypes. Although the majority of published single-cell studies of RCC TIL have focused on gene expression and phenotypes, three small studies with a total of 8 RCC subjects (11, 41, 43) did combine TCR repertoire analysis with gene expression. In these studies, expanded T cell clones were also present in each of the five tumors studied, consistent with our observations.

The expanded clonotypes present only in tumor preferentially displayed a T_em_ phenotype with the highest expression of antigen-activation and exhaustion markers of any T-cell subset within the TME. Previous studies have suggested that exhausted, antigen-experienced CD8^+^ T_em_ cells within tumor may be derived from TCF1^+^CD28^+^ stem-like T cell (T_scm_) precursors that localize to antigen presenting cell-rich niches within tumors (44). A subset of the tumor-restricted expanded clonotypes expressed the cell cycle marker Ki67 suggesting *in situ* T-cell clonal expansion within the TME consistent with the stem cell origin model. Importantly, our IHC studies showed that Ki67^+^ T cells were not uniformly distributed within tumors and co-localized with areas of viable tumor suggesting a directed homing of tumor-antigen reactive T-cell clones towards target antigen presented on viable RCC tumor cells. A subset of the T_em_ clones was found to comprise CD4^+^CD8^+^ double positive T cells that shared a similar antigen-experienced phenotype with high level expression of exhaustion markers as for CD8^+^ single positive clones. Our pathway enrichment analysis indicated these cells, when compared with CD4**^+^** or CD8**^+^** single positive T-cells, have upregulated the expression of genes in pathways involved in MHC antigen presentation, TCR signaling, and PD-1 signaling. This result is consistent with the published studies reporting that double positive T cells with an antigen-experienced phenotype have most frequently been observed in RCC, other urological cancers, and melanoma (38, 45, 46). However, the functional significance of CD4^+^CD8^+^ co-expression in subpopulations of RCC TIL is currently unknown.

The clonotypes shared among tumor, NAT, and blood preferentially displayed a T_eff_ phenotype expressing higher levels of cytotoxic markers but lower levels of activation and exhaustion markers than the tissue-restricted clonotypes. This subset included the largest individual clonal populations, and the continued circulation of these expanded clones was maintained in peripheral blood of one patient for at least a year. These clonotypes were also associated with upregulation of a transendothelial migration signature as well as the pro-inflammatory chemokine receptor *CX3CR1*. Our data suggest that the fractalkine receptor *CX3CR1*-expressing CD8^+^ Teff cells circulate in blood and may serve as a reservoir to replenish T cells within the tumor. Further studies of the determinants of T-cell entry into the tumor, especially the phenotype of tumor endothelial cells, may provide insights to improve T-cell entry into the TME.

Although we observe that most T cells isolated from RCC tumors demonstrate the hallmarks of antigen encounter (47), our studies have not yet generated direct evidence for recognition of RCC tumor cells by RCC TIL. High-throughput platforms for TCR antigen discovery are not readily available, and represent a priority area for future investigation for our research group. Moreover, in view of the evidence for persistence circulation of expanded TCR clonotypes in some patients, ignorance of the T-cell antigen targets on RCC tumor limits the potential to interrogate antigen loss as a mechanism of tumor escape from immune control (48). Of interest, we report here the discovery of four “public” TCR clonotypes that are shared by 10 to 14 RCC patients in a 53-patient cohort. These clonotypes are carried by CD8^+^ T cells that display both the tumor-restricted, T_em_ phenotype, and the shared T_eff_ phenotype. These TCRs will be prioritized for antigen discovery efforts by our research group, as an easily identifiable public clonal TCR response would be an invaluable tool to provide insight into the behavior of tumor-specific clonotype responses in RCC patients treated by ICI-based regimens and may identify TCRs suitable for therapeutic manipulation by vaccination or engineered transgenic T-cell techniques.

As a surrogate for antigen discovery, we deployed the GLIPH2 algorithm for clustering TRB-CDR3 amino acid sequences based on predicted shared antigen specificity. This analysis revealed a consistent finding of less complex specificity groupings in RCC tumor than found in blood or NAT-derived TCR repertoires. These data suggests a heretofore unrecognized control point for T-cell entry into the TME. Inspired by the observation by our group and others of marked heterogeneity in T-cell density both within and between RCC tumors, our group is focusing significant efforts on understanding the potential role that RCC-associated vascular endothelium may play in influencing the spatial heterogeneity of T-cell trafficking into the TME (49, 50).

In summary, our observations based on transcriptomic sequencing and CDR3 tracking at the single cell level suggest a model for two distinct populations for the most abundant, clonally expanded T cells in the ccRCC microenvironment: 1) local antigen encounter and clonal expansion within the TME; and 2) CD8^+^ T_eff_ cells trafficking from blood that may serve as a pool to replenish T cells in the TME (11). Future research will be directed to understand the determinants of the spatial regulation of intra-tumoral T-cell expansion as well as T-cell trafficking into the TME. This framework can be extended by studying larger numbers of RCC patients treated with various immunotherapy regimes including ICIs to further explore the prognostic and therapeutic impact of these two populations. Our work also suggests complexities for *ex vivo* TIL expansion for autologous cellular therapy that may reveal distinctions between these clonal populations in patients with different therapeutic outcomes.

## Data availability statement

The TRB repertoire sequencing, 10X, and targeted scRNAseq datasets presented in this study can be found in online repositories. The names of the repository/repositories and accession number(s) can be found below: https://github.com/YuexinXu/RCC_CLIP_Data, RCC_CLIP_Data.

## Ethics statement

The studies involving human participants were reviewed and approved by the Institutional Review Board at Fred Hutchinson Cancer Center. The patients/participants provided their written informed consent to participate in this study.

## Author contributions

ST, EW and YX designed the study. YX, AM performed the experiments. AT performed the *TRB* sequencing. YX performed the data analysis. All authors interpreted the data. YX, ST wrote the manuscript. CM, SA, and EW reviewed and revised the manuscript. All authors approved the final version.

## Funding

This research was funded by the Clinic and Laboratory Integration Program of the Cancer Research Institute, the Kidney Cancer Research Program from Department of Defense (W81XWH19-1-0789), a Young Investigator Award from Kidney Cancer Association (FP50006666), and generous support from philanthropic donations. This research was also supported by the Flow Cytometry, Genomics, Bioinformatics, Immune Monitoring, and Experimental Histopathology Shared Resources of the Fred Hutch/University of Washington/Seattle Children’s Cancer Consortium (P30 CA015704). We thank the high-performance computing team at the FHCRC for providing scientific computing infrastructure funded by a National Institutes of Health Office of Research Infrastructure Programs grant S10OD028685.

## Acknowledgments

We thank the patients and their families for participating in the research.

## Conflict of interest

The authors declare that the research was conducted in the absence of any commercial or financial relationships that could be construed as a potential conflict of interest.

## Publisher’s note

All claims expressed in this article are solely those of the authors and do not necessarily represent those of their affiliated organizations, or those of the publisher, the editors and the reviewers. Any product that may be evaluated in this article, or claim that may be made by its manufacturer, is not guaranteed or endorsed by the publisher.

## Abbreviations

CAIX, Carbonic anhydrase IX; ccRCC, clear cell renal cell carcinoma; CDR3, complementarity determining region 3; FFPE, formalin-fixed and paraffin-embedded; ICI, immune checkpoint inhibitor; IHC, immunohistochemistry; mIHC, multiplex immunohistochemistry; NAT, normal adjacent tissue; PBMC, peripheral blood mononuclear cell; RCC, renal cell carcinoma; scRNAseq, single-cell RNA sequencing; T_cm,_ central memory T cell; Th1, type 1 helper T cell; Th2, type 2 helper T cell; Th17, type 17 helper T cell; T_eff_, effector T cell; T_em_, effector memory T cell; T_reg_, regulatory T cell; T_rm_, residential memory T cell; T_scm_, stem-like memory T cell; TCR, T-cell receptor; TIL, tumor-infiltrating (T) lymphocyte; TME, tumor microenvironment; tSNE, t-distributed stochastic neighbor embedding.
