# Supplementary material for: Integrated TCR repertoire analysis and single-cell transcriptomic profiling of tumor-infiltrating T cells in renal cell carcinoma identifies shared and tumor-restricted expanded clones with unique phenotypes
Source: Front Oncol. 2022 Sep 14;12:952252. doi: 10.3389/fonc.2022.952252 (PMC9515957; doi:10.3389/fonc.2022.952252)
Supplement: Supplementary file 1 [file DataSheet_1.docx]

Supplementary Material

# Supplementary Figures and Tables

##
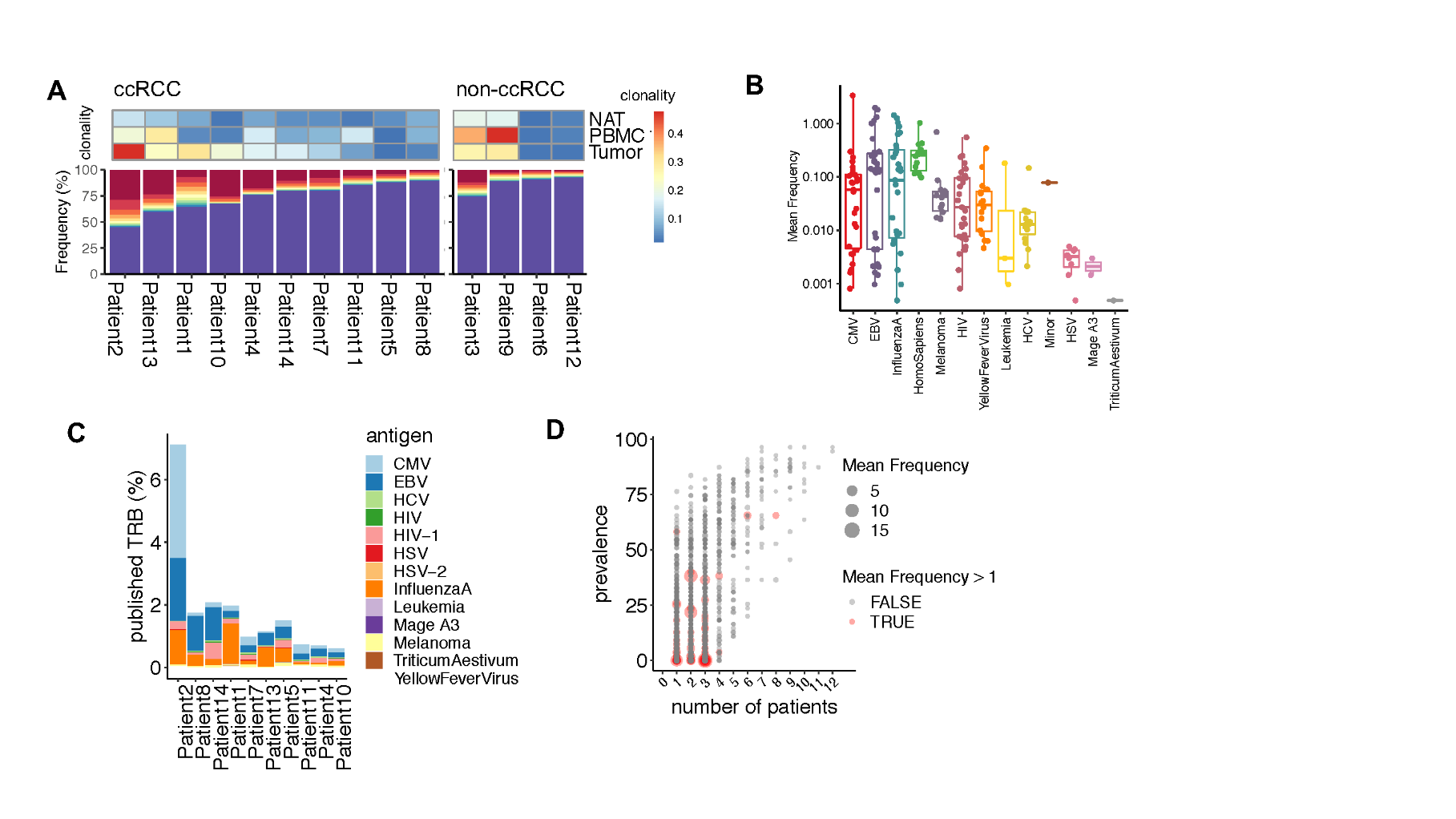
Supplementary Figures

**Supplementary Figure 1.** **Clonality and antigenic specificity of TRB repertoires in clear cell and non-clear cell RCC. (A)** Top: Clonality of TRB repertoires in NAT, PBMC, and RCC tumor from 10 subjects with ccRCC and 4 with non-ccRCC. The color represents numeric values of clonality in NAT, Tumor and PBMC. Bottom: Frequency distributions of the 10 most frequent TRB CDR3 sequences in the 14 RCC tumors. The 10 rainbow-colored segments at the top of each column represent the 10 most frequent TRB CDR3 amino acid sequences in the 14 RCC repertoires; the purple segments represent the balance of each RCC TRB repertoire. **(B)** Frequency of TRB CDR3 amino acid sequences in RCC TIL with reported associations with CD8**^+^** or CD4**^+^** T-cell responses to specific pathogens, cancers, or tissues. **(C)** Frequency distribution of TRB CDR3 sequences in RCC TIL repertoires of 10 subjects with reported specificity for the indicated pathogens, cancers, or tissue antigens. **(D)** Sharing of specific TRB sequences (each represented by a dot) in RCC TIL across 14 patients (x-axis) plotted against the prevalence of each sequence in the PBMC TRB repertoires of 55 control adults without cancer.


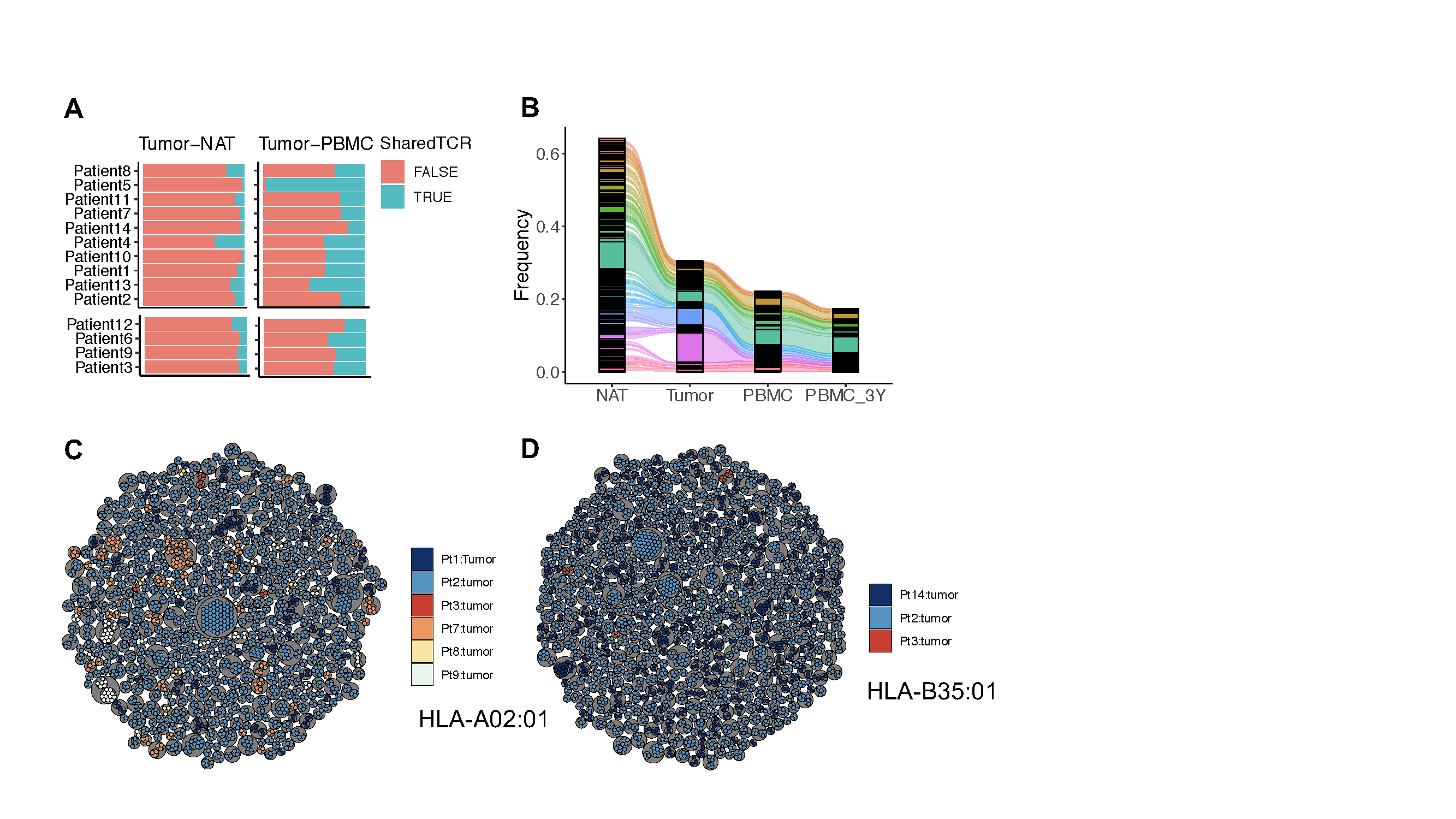


**Supplementary Figure 2. TRB sequences and specificity group sharing within patients and across patients.** **(A)** Proportion of TRB sequences in the TIL repertoires of 10 participants with ccRCC (top) and 4 participants with non-ccRCC (bottom) that are also detected (teal, “true”) in the autologous NAT (left) or PBMC (right) repertoires. **(B)** Alluvial plot of the frequency distribution of TRB sequences observed at > 0.1% frequency in the RCC TIL of participant 7 in autologous NAT or PBMC at the time of resection or in PBMC 3 years after resection. Colored rivulets identify sequences shared by two or more tissues. **(C)** and **(D)** T-cell packing plots of GLIPH2-predicted antigenic specificity groups in the TRB repertoires of participants sharing expression of the class I MHC alleles HLA-A*02:01 (C; 6 participants) or HLA-B*35:01 (D; 3 participants). Small circles represent individual TRB sequences and larger circles indicate specificity groups.


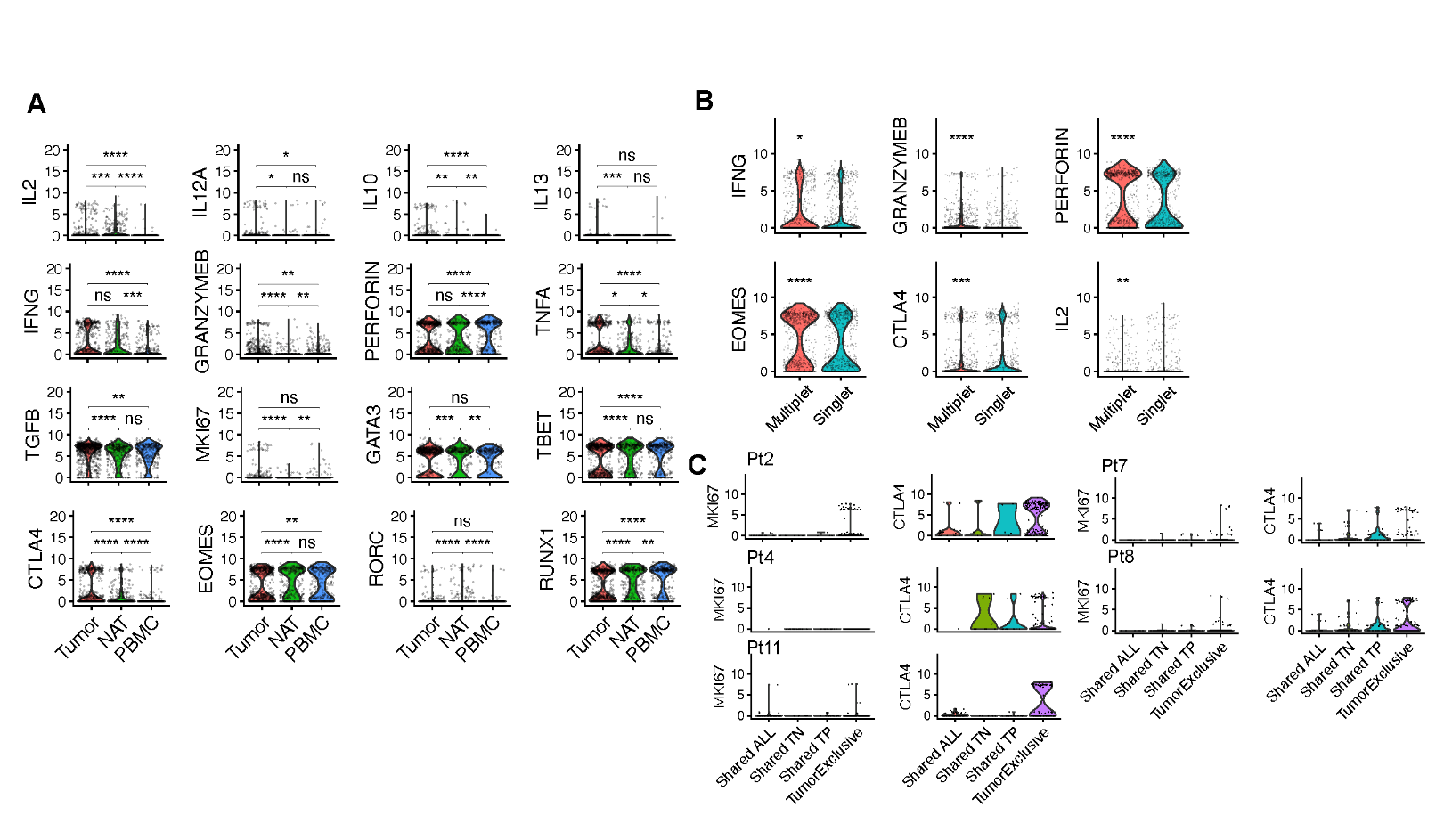


**Supplementary Figure 3. Targeted scRNAseq from six participants**. **(A)** Violin plots of the expression of selected genes included in the targeted sequencing panel in T-cells in RCC tumor, NAT, and PBMC. The log_10_ gene expression was plotted for each single cell (dot event).  **(B)** Expression of the selected genes in multiplets or singlets. Multiplet, multiplet tumor-infiltrating T cells, a *TRA*/*TRB* sequence pair detected more than once in a specific tumor sample; Singlet, singlet T cells, a *TRA/TRB* sequence pair detected only once in tumor. **(C)** Expression of *MKI67* and *CTLA4* in *TRA*/*TRB*-marked T-cells with the indicated patterns of tissue distribution (ALL: RCC tumor, NAT, and PBMC; TN: RCC tumor and NAT; TP: RCC tumor and PBMC; or RCC tumor only) in five participants with ccRCC. ** P ≤ 0.01, *** P ≤ 0.001, **** P ≤ 0.0001, *t*-test.


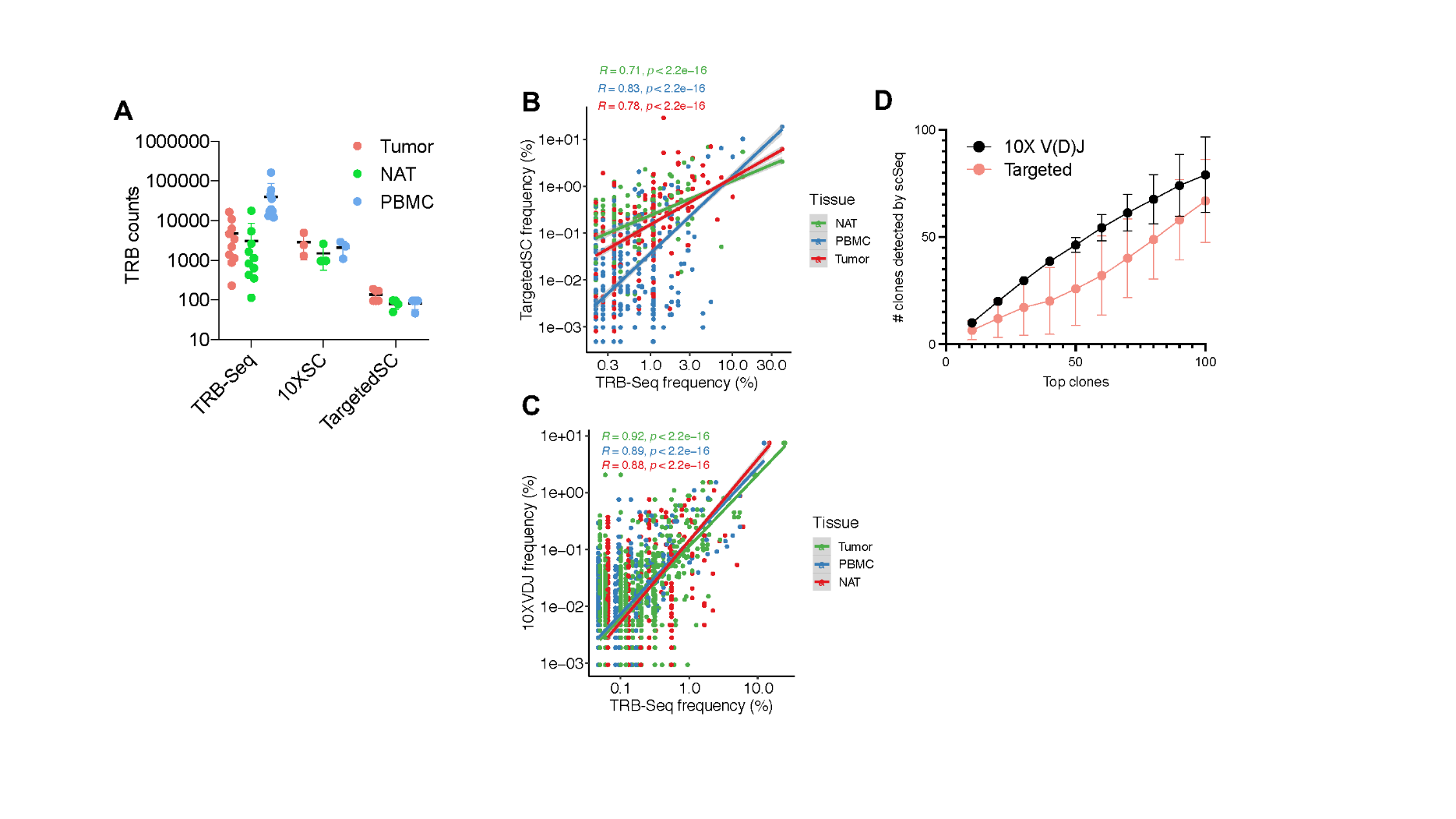


**Supplementary Figure 4. Comparison of deep TCR repertoire sequencing, targeted and whole transcriptome scRNAseq. (A)** Unique TRB CDR3 amino acid sequences recovered from RCC tumor, NAT, and PBMC samples across three sequencing platforms: TRB repertoire sequencing, 10X Genomics scRNAseq, and targeted scRNAseq. Each data point represents a biological sample. **(B and C)** Correlation between the frequency of specific TRB sequences in NAT, PBMC, and RCC tumor as inferred from **(B)** population TRB sequencing and targeted scRNAseq or **(C)** population TRB sequencing and 10X Genomics scRNAseq.  **(D)** Comparison of the efficiency with which the most frequent TRB sequences identified using population TRB sequencing were also identified using targeted scRNAseq (rose) or 10X Genomics scRNAseq (black).


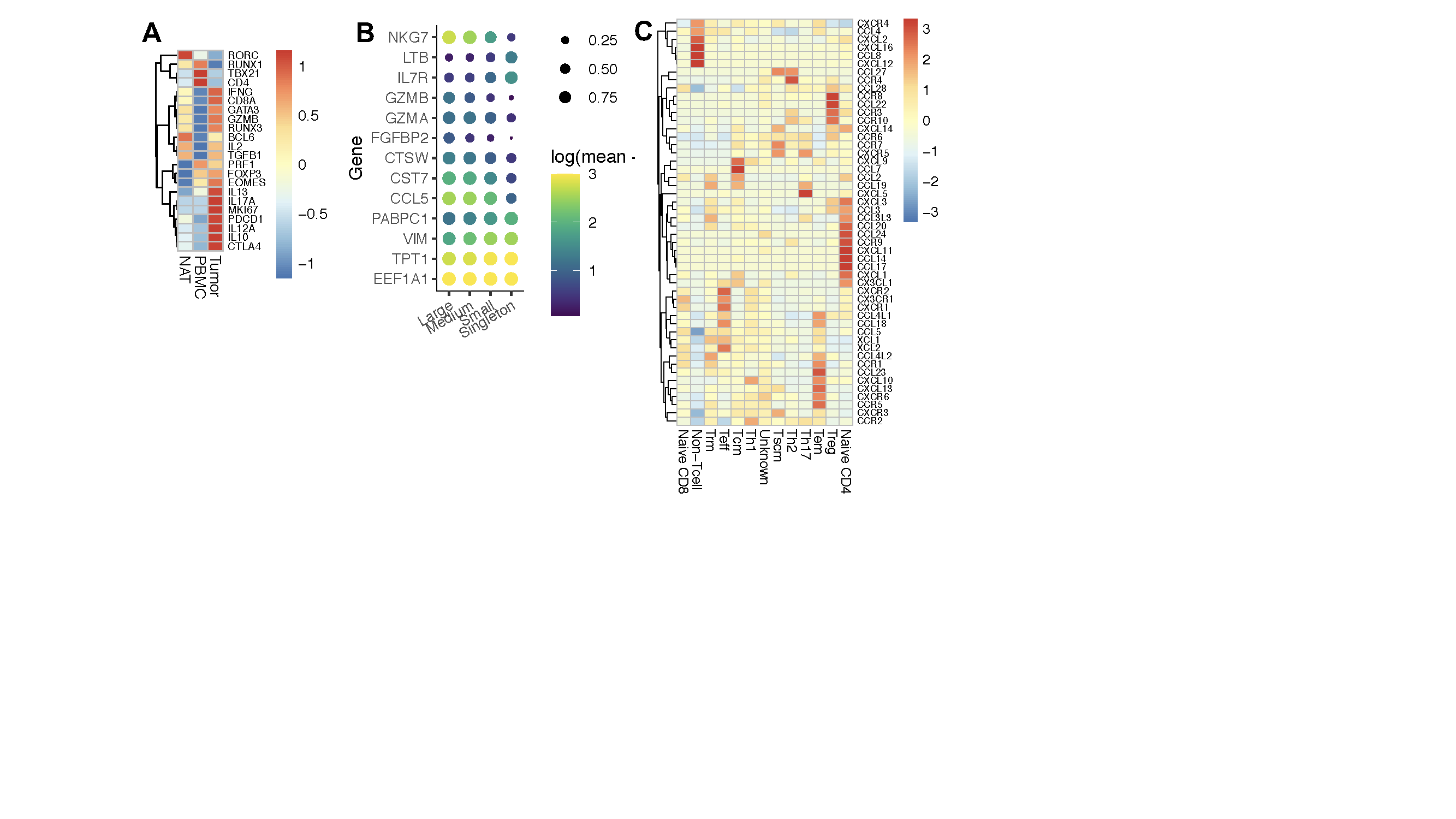


**Supplementary Figure 5. Gene expression in different subtypes of T cells**. **(A)** Relative expression of selected genes in CD3**^+^** T cells carrying unique TRA/TRB amino acid pairs and found in NAT, PBMC, or RCC tumor. **(B)** Relative expression of selected genes in CD3**^+^** T cells carrying unique TRA/TRB pairs and classified according to clonal size (large, medium, small, or singletons). **(C)** Relative expression of chemokine / chemokine receptor genes from the KEGG signaling pathway (hsa04062) in T cells from ccRCC tumors classified according to their inferred phenotype.

**Supplementary Figure 6. Clonal architecture and enriched pathway of tumor-infiltrating double positive T cells**. **(A)** T cell packing plot of double positive T cells in RCC tumors. Large dark grey circles represent unique clonotypes in ccRCC tumors, and small circles represent individual cells harbor the same clonotype. Red colored inner circles indicate cells that are CD4^+^CD8^+^, grey indicate T cells that are single positive. **(B)** Enriched pathways in tumor-infiltrating CD4^+^CD8^+^ double positive T cells compared with CD8^+^ single positive T cells. **(C)** Enriched pathways in tumor-infiltrating CD4^+^CD8^+^ double positive T cells compared with CD4^+^ single positive T cells.

## Supplementary Tables

**Table S1. Targeted scRNAseq phenotype primers**

**Table S2. Garnette markers for cell classification**
